# A Web-Based Self-management App for Living Well With Dementia: User-Centered Development Study

**DOI:** 10.2196/40785

**Published:** 2023-02-24

**Authors:** Abigail Rebecca Lee, Emese Csipke, Lauren Yates, Esme Moniz-Cook, Orii McDermott, Steven Taylor, Michael Stephens, Daniel Kelleher, Martin Orrell

**Affiliations:** 1 Institute of Mental Health School of Medicine University of Nottingham Nottingham United Kingdom; 2 Division of Psychiatry University College London London United Kingdom; 3 Department of Clinical, Educational and Health Psychology University College London London United Kingdom; 4 Faculty of Health Sciences University of Hull Kingston-upon-Hull United Kingdom; 5 Ayup Digital Leeds United Kingdom; 6 Research & Development Humber Teaching National Health Service Foundation Trust Willerby United Kingdom; 7 Centre for Applied Dementia Studies University of Bradford Bradford United Kingdom

**Keywords:** dementia, self-management, independence, quality of life, web-based, website, psychosocial

## Abstract

**Background:**

Self-management, autonomy, and quality of life are key constructs in enabling people to live well with dementia. This population often becomes isolated following diagnosis, but it is important for them to feel encouraged to maintain their daily activities and stay socially active. Promoting Independence in Dementia (PRIDE) fosters social inclusion and greater dementia self-management through an interactive handbook.

**Objective:**

This study aimed to develop a paper-based PRIDE manual on a web-based platform.

**Methods:**

Two overarching stages were used to create the web-based version of PRIDE. The first was *Preliminary Development*, which encompassed tendering, preliminary development work, consultations, beta version of the website, user testing and consultation on beta version, and production of the final web-based prototype. The second stage was *Development of the Final PRIDE App*, which included 2 sprints and further user testing.

**Results:**

Through a lengthy development process, modifications were made to app areas such as the log-in process, content layout, and aesthetic appearance. Feedback from the target population was incorporated into the process to achieve a dementia-friendly product. The finished PRIDE app has defined areas for reading dementia-related topics, creating activity plans, and logging these completed activities.

**Conclusions:**

The PRIDE app has evolved from its initial prototype into a more dementia-friendly and usable program that is suitable for further testing. The finished version will be tested in a reach, effectiveness, adoption, implementation, and maintenance study, with its potential reach, effectiveness, and adoption explored. Feedback gathered during the reach, effectiveness, adoption, implementation, and maintenance study will lead to any further developments in the app to increase its applicability to the target audience and usability.

## Introduction

### Background

Living well with dementia has often been constructed around quality of life, choice, autonomy, dignity, and staying as independent as possible [1]. People with dementia have identified how they quantify living well, which included involvement at home and in the neighborhood, independence, self-management of symptoms, and quality of life. They also recommend that these should be considered when developing dementia-specific interventions [2]. Many people with dementia have the ability to maintain an active and social life, but some of the negative effects of receiving a diagnosis, depression, or diagnosis stigma can result in social isolation and withdrawal from society [3,4]. It is important that people living with mild dementia are supported and encouraged to maintain their normal activities, remain independent, and stay active within society for as long as they are able to.

Promoting Independence in Dementia (PRIDE) is a psychosocial program designed for people living with mild dementia, whose symptoms of dementia affect day-to-day activities, but are able to live relatively independently and to promote choice, autonomy, and social inclusion. It encourages them to maintain and develop cognitive, physical, and social activities to improve their self-management, independence, and quality of life. The content is delivered in a manualized format, with interactive activities and discussion points, such as creating activity plans. Users are paired with trained facilitators who go through the PRIDE program and support the development and execution of personalized activity plans. Across 3 sessions, users and facilitators plan, carry out, and review users’ individual plans and discuss how techniques learned through PRIDE could support them in approaching activities in the future.

A multicenter feasibility study of the PRIDE program provided participants with both a paper manual and an electronic version, so they were able to choose whether to use one or both formats [5]. The paper manual was the most popular, being used by all participants in the intervention arm, but 1 participant chose to use both the paper and the electronic versions. The findings suggested that the PRIDE intervention was a useful and relevant program to promote independence and support people with dementia in their daily activities, and it was generally well-received by the participants [5]. Although only 1 participant accessed the electronic version of PRIDE, the COVID-19 pandemic meant that more people have resorted to web-based resources; therefore, further developments to refine the PRIDE web-based app would enable it to reach those who have become further isolated during the pandemic and beyond [5].

This type of intervention delivery has the potential to be successfully adopted by people with dementia and their families [6], but little is known about the technological processes required to develop high-quality web apps for people with dementia and their families. However, more high-quality research is needed in this area, including more consideration of the barriers to and facilitators of use and how these impact adoption.

### Aim

As part of a large research program, a paper-based manual psychosocial intervention for the PRIDE program was developed and tested for feasibility [5]. Here, we describe the processes associated with the technological work and adaptation of the manualized PRIDE intervention into a usable web-based platform, the PRIDE app. The aims of the web platform development were to (1) design an innovative log-in system tailored to the needs and abilities of people with dementia and (2) involve project stakeholders in the development of the website to ensure that the intervention is tailored to their needs, preferences, and abilities. This involvement would help involve more consideration of the barriers to and facilitators of use for the PRIDE app.

## Methods

**Figure 1:**
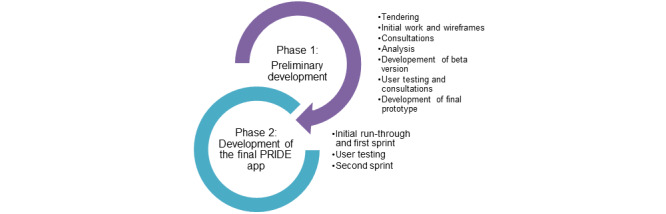
Outline of the 2 development phases of the Promoting Independence in Dementia (PRIDE) app. Each phase included multiple substages of work.

Figure 1 outlines the 2 development phases involved in creating the web-based PRIDE.

### Phase 1: Preliminary Development

#### Overview

Work on the development of the PRIDE website began upon completion of the second draft of the PRIDE intervention [7] and ran concurrently with feasibility testing of the paper-based version of the program. The development stages of the web-based platform were (1) technological work, including project tendering and preliminary development; (2) consultations; (3) development of a beta version of the website and user testing or consultation; and (4) production of the final web platform.

#### Technological Work: Tendering and Preliminary Development Work

An invitation to tender was written with input from MindTech Healthcare Technology Co-operative, a National Institute for Healthcare Research–funded national center for the development, adoption, and evaluation of new technologies for mental health care and dementia. The standard university tendering procedure managed by the procurement department was followed. Developers accessed the brief, which included details of the PRIDE intervention and requirements from the web app (eg, must be user-friendly and adhere to Dementia Empowerment Engagement Programme guidelines [8]), and bid for the work contract.

In total, 26 bids were received, and 2 members of the PRIDE team independently reviewed all bids and rated them according to the standardized scoring criteria provided by procurement. The dimensions of the bids assessed included service delivery, website development, implementation plan, and data security. Each dimension received a pass or fail, and notes were made to support these ratings. A total quality score was generated based on scores from each dimension; bids were ranked, and a shortlist was made, which was reviewed by a Digital Research Specialist. The final shortlist (7 bids) was further discussed, the outcome of which was the selection of 4 software companies to be interviewed. Ayup Digital Designs was commissioned to do the work on the basis of the demonstration of an excellent understanding of the intervention, dementia-friendly design, and previous experience in health and social care–based projects.

A user-centered design approach, broadly in line with the Government Digital Service Standard Agile Delivery methodology, was used. A *discover* meeting attended by representatives from Ayup and the PRIDE team occurred to consolidate the company’s understanding of the intervention and discuss ideas for how the paper-based manual content and processes of the intervention would be adopted for the website. Ayup conducted work on information architecture, user journeys, user experience, and interface design. Alpha-stage wireframe designs were created and reviewed. The work outputs facilitated further discussion of how the website would work in practice (eg, how information would be navigated and presented by stakeholders).

#### Consultations

Multiple consultations were arranged during iterations of the website. An opportunistic sample of key stakeholders was invited to discuss the initial drafts, including log-ins, fonts, colors, and layout. Then, 3 consultations were held. The first group comprised 3 members of the University of Nottingham (UoN) Patient and Public Involvement (PPI) dementia group, which met regularly, typically attended by people with dementia, caregivers, researchers, representatives from local community organizations, and health care professionals. Participants were invited to participate in consultations following a presentation on the PRIDE project. They had not participated in any aspect of PRIDE. Members of the PPI group with dementia were actively and regularly involved in PPI, community, and research activities associated with universities and other organizations, such as the National Health Service; therefore, their participation in these consultations was not considered above and beyond their usual activities.

The second consultation involved a person with dementia, their supporters, and memory nurses who had participated in the PRIDE feasibility study. These participants had insight into the experience of receiving or delivering the intervention in practice; therefore, they could comment in depth on the content of the intervention and intervention processes and directly compare the paper-based and web app versions. The memory nurses invited dyads (people with dementia and their supporters) who had completed or were part of the intervention in the session. A third consultation was conducted via teleconference with a researcher who had delivered several intervention sessions using the paper manual and materials at a PRIDE site. The lead PRIDE researcher, who provided support and training, contacted the researcher via email with an invitation to participate.

Consultations were planned to last for a maximum of 3 hours. Examples of website wireframes (blueprints that show the basic framework of a website) were shown on a projector screen with pages adjusted for size as necessary. Before the end of the discussion, the web developer summarized the key points from the notes and asked the group to confirm if these reflected their comments. The second consultation was shorter, lasting 2 hours. A web-based videoconferencing program was used for the third, so that the wireframes could be viewed.

#### Discussion Topics

Discussions in the first consultation focused on (1) the use of technology to identify which devices the intervention would most likely be accessed on (eg, tablet, mobile, or laptop); (2) challenges with technology to highlight user experience; (3) the PRIDE log-in system to determine whether the innovative methods proposed were acceptable to stakeholders (easy to remember yet secure); and (4) feedback for a limited selection of wireframes and examples of design features (eg, font, color palettes, and icons) that were shown to the group.

The same discussion points were covered by participants in the second consultation, but participants were also asked how best to adapt the paper-based version of the program for delivery via the website. The group considered proposed ideas for the website presentation of activities featured in the paper-based materials (eg, completion of the profile) and how the website could be used to facilitate interaction among the person, supporter, and facilitator during the session compared with the paper-based manual and worksheets. The third consultation focused on reviewing the wireframes and considering the functionality of the website from the perspective of an interventionist with experience in delivering PRIDE.

#### Analysis

Notes were taken at consultations by the researcher facilitating the session and the website designer. These were circulated among the teams and collated after the consultation. No formal analyses were performed on the gathered data; however, action points were generated for use by the website developer to create further versions of the website wireframes.

#### Development and User Testing of the Beta Version

Findings from user research activities were synthesized, and assumptions about user stories or website features were tested and validated. A further round of design iterations was undertaken before a beta version of the website was developed.

The beta version was reviewed by the research team and checks (eg, spelling, grammar, and flow through the intervention process) were performed, and consultation sessions were arranged with stakeholders. The purpose of these consultations was to observe participants using the website and gather comments on usability issues such as ease of navigation. A key aspect of user testing was to enter dummy data into the activity sections of the website and set up of the log-in system.

Consultations on the beta version of the website included 4 individuals with dementia, 4 supporters, 2 PPI members, and 3 intervention facilitators. Consultations took place at the homes of consultees, in the National Health Service, or in the university departments. Researchers were provided with a topic guide, including questions, prompts, and a list of tasks for consultees to complete (eg, logging in and out of the website). Researchers implemented a “think-aloud” protocol, encouraging consultees to comment as they used the website to yield insight into their experience, particularly areas of difficulty [9]. Comments were noted and supplemented with notes written by the researcher.

Feedback from user testing and consultations was given to the design team, who subsequently made design tweaks to the beta version to enhance usability. The full website was developed with special attention to ensure the website was as accessible as possible.

#### Informed Consent and Ethics Approval

All consultations were informal, where no personal information about the participants was collected and the discussions were not recorded. All participants provided verbal consent to participate in the discussions. Consultations were specified in the PRIDE protocol, based on which the study received ethics approval from East Midlands Nottingham Research Ethics Committee (16/EM/0044). All participants with dementia were in the early stages of the condition and were deemed to be able to provide verbal consent for their involvement by the recruiting researcher.

### Phase 2: Development of the Final PRIDE App

#### Overview

Researchers and Ayup agreed on continuing an agile approach to app development, as it enabled dynamic collaboration between all relevant stakeholders and was also the standard practice for Ayup. As part of this approach, intensive development periods called sprints were incorporated to ensure priority work was completed within a specific timeframe. For this stage of development, each sprint would last 1 week, and Ayup’s workload would be specifically aimed at the PRIDE app.

#### Initial Run-through and First Sprint

The work on further developing the PRIDE app began in November 2019. An initial run-through of the prototype was conducted by 2 researchers at the UoN (ARL and OM), with a list of issues regarding the design, functionality, and content of the web app collated. One researcher viewed the app from a practical viewpoint, whereas the other used their knowledge and experience of working with people with dementia and viewed it from their perspective. The potential amendments were noted and discussed by the study team. A specification document was compiled and sent to Ayup, the company responsible for app development for the study. Following the initial run-through, 2 development sprints were scheduled for spring and summer 2020.

The focus for the first sprint was the highest priority issues identified with regard to the functionality, content, and overall design of the PRIDE app. Specification and priority documents were supplied to Ayup before a sprint planning meeting between the study team and development company. This provided the opportunity to discuss the workload and clarify any final improvements before the sprint start date.

#### Specification and Priority Documents

The specification document outlined the following goals and key points for the first sprint:

- Navigation to and between sections—clearer signposting of the content, such as the addition of a contents page, so users can see which section they are completing, and making the sidebar menu items more evident
- Larger font and better page layout (less empty white space)—reduction in the amount of text per page to reduce the need to scroll down the screen and increase in font size
- Addition of identifiable icons—clear and consistent use of easily recognizable icons, with particular attention given to the navigation icons including “Home,” “Help,” and “Back”
- Maintained access to introductory session content—the prototype did not allow users to revisit sessions from the first intervention session

Priority tasks were identified as fundamental, high, or low using the MoSCoW (Must Have, Should Have, Could Have, and Will Not Have this time) prioritization framework. The target was all fundamental and high-priority tasks to be completed within the first sprint. Fundamental tasks included enabling continued access to introductory session content, increasing font size, addition of show or hide tabs to reduce long sections of text, and improvements to navigation and signposting. High-priority tasks included the addition of activity icons and instructions, inclusion of a glossary link on the user’s main dashboard, and fixing graphical glitches on images. The sprint was completed in April 2020, with all fundamental and high-priority tasks being completed. Those tasks that were of lower priority were held over to the second sprint.

#### User Testing

Following completion of the first sprint, it was important to obtain feedback from the target user group. Contact was made with established PPI groups at the UoN and in the local community with the aim of recruiting volunteers to provide “expert consultations.” Adverts for volunteers were posted on various social media feeds. The Alzheimer’s Society was also contacted but was unable to publicize the call for volunteers because of the COVID-19 pandemic. Two volunteers, a person living with young-onset dementia and their partner who were members of an established PPI group, were recruited for the user-testing stage. Written guidance on accessing and navigating the PRIDE app was provided, and the volunteers could contact the team if they encountered any problems. The volunteers explored the PRIDE app in their own homes over the course of a week before providing written feedback on their experiences.

Overall, the feedback gathered consisted of a mixture of positive and negative comments. The log-in process was perceived as easy to use, and the activities prompted positive discussions between users. However, they did think that some of the content was aimed at older adults with dementia, rather than young-onset dementia, and therefore might not be as relevant to those of all ages living with dementia. They also found that working slowly through each section and making notes helped people with dementia follow the content. Feedback from this stage was actively provided during the second sprint stage. Some comments from the user testing are as follows:

Logging in was straightforward.

The plan, do, review process made sense to [the person with dementia] when I worked through it with him and prompted ideas for things that would help/hinder him in the activities he wanted to try doing.

Impact of COVID on going out and socializing might need to be factored in.

Generally, [the person with dementia] found it difficult to tackle more than a few sections in one sitting. When we started work the next day, he had forgotten what he had done previously. We found working through each section slowly and making notes or drawing something to reflect our conversations made things easier.

#### Second Sprint

A second sprint was originally planned for the summer but owing to the difficulties in finding user-testing volunteers and the impact of the COVID-19 pandemic, the sprint was delayed until September 2020. The focus was on making the improvements and amendments identified during the user-testing stage. Similar to the first sprint, a specification document was sent to Ayup with developmental changes before the start date. For this sprint, the document highlighted grammatical errors that needed to be resolved in the content; identified words and phrases that could be changed to increase clarity and make the content more dementia-friendly; and added a paragraph regarding the impact of the COVID-19 pandemic and how this could affect their activities. This information was also uploaded to Trello, a planning software, which enabled us to prioritize actions and estimate the time taken to complete these actions. This allowed a more collaborative approach to sprint work between the study team and Ayup, and the researchers were able to monitor the progress of tasks during the sprint. All high- and medium-priority changes were made, such as correcting typographical and grammatical errors and adding a statement about how the COVID-19 pandemic could affect the ways in which people use the PRIDE app, which vastly improved the usability and functionality of the PRIDE app, bringing it up to a standard suitable for use by participants.

Before the COVID-19 pandemic, a field-testing stage was planned to follow the completion of the second sprint. Volunteers would have completed a remote run-through of the PRIDE app with the study team and provided additional feedback on the app’s usability and functionality from the perspective of the target population. However, owing to difficulties in recruitment experienced during the user-testing stage and the additional constraints and impact of the pandemic, this stage was removed.

## Results

### Phase 1: Preliminary Development

#### Overview

On the basis of the discussions of previous research on how people with dementia may use technology and their specific needs, an initial draft of the wireframes was developed. It was important for Ayup to understand the range of stakeholders’ digital literacy and the ability to best design an experience that meets their needs.

Keeping in mind the deterioration in cognitive skills characteristic of dementia, the team developed a log-in system that would not require the user to remember a password but that would uniquely identify their account and uphold security. Ayup proposed that an intervention facilitator assigned to a person with dementia create a PRIDE account for them in the first instance, which consists of basic data including name, date of birth, and contact details. This becomes their “PRIDE profile.” Once an account is created, the person can log in to the PRIDE website by entering their initials and date of birth; then, a unique, single-use 4-digit code is sent to a registered contact number via an SMS text message or an automated telephone voice message.

#### Log-in Process

The concept of the log-in system was discussed with consultees to determine its acceptability. Consultees with dementia in groups 1 and 2 acknowledged that dementia may affect their ability to remember passwords. They described “fear” of having passwords, feeling the information was too important to lose if forgotten, and identified potential safety risks of strategies to remember passwords such as writing them down. The idea of a log-in system using initials, date of birth, and a single-use code was well accepted. Although the date of birth relies on memory, a consultee said that this information is a personal possession and something that never changes, so they thought it would be difficult to forget. One consultee described feeling “lucid” and able to solve problems using logic at the moment, for instance, to navigate the log-in system but that they did not know how long they would be able to do this. Consistent with this concern, a consultee in group 2 suggested the use of the proposed log-in system; there would have to be instructions on the screen to remind the person of the sequence to follow.

In contrast, intervention facilitators participating in consultations 2 and 3 suggested having log-in details saved in a browser might be a simple way to assist people to remember passwords without the need for a specific log-in system. However, people with dementia in consultations 1 and 2 were wary of saving passwords automatically through their web browser or using autofill functions, as they felt this was less secure and anyone could potentially access their personal information. When asked whether it would be preferable to receive the single-use code via telephone call or SMS text message, many consultees said that the telephone call may be a problem, as they have call-screening devices to prohibit unknown or nuisance calls. Some said that as long as they knew that they would be receiving the call, they could pick it up. The final log-in system uses a combination of initials, date of birth, and a single one-time code that is either sent via SMS text message or via telephone using text-to-speech technology.

#### Paper-Based Materials Versus a Web-Based Platform

Consultees in group 1 had not previously taken part in the PRIDE intervention but were asked if they would have a preference for paper-based or web-based materials if they were to take part in PRIDE. Two said it would be easier to use paper-based materials, adding that they did not have to think about things going wrong with technology. The person with dementia and supporters in consultation 2 who had used paper-based materials in the feasibility study preferred the website format, identifying the following benefits: (1) it would be easier for intervention facilitators to see necessary information (eg, plans) on the web rather than having to refer to several sheets of paper; (2) it would be a more effective way of delivering reminders instantly as you might forget to look at a calendar; (3) it might stimulate the person and lead to uptake of other activities such as brain training that might be helpful; and (4) it might be easier to read typed text and type text than to read and write for people with dementia.

Supporting the first point, intervention facilitators in consultation 2 added that they had experienced problems with people losing the manual and paperwork between sessions, and if the supporter was not present in sessions 2 and 3, it was difficult to determine what had actually been done without the accompanying paperwork. The intervention facilitator in consultation 3 felt the website wireframes seemed to relate to the paper-based manual quite well.

#### Concerns About Technology

Intervention facilitators stated that in their experience, many older people with dementia did not use or have computers but many used mobile phones or had computer tablets. Intervention facilitators said that the use of computers would depend on the age group and raised the point that some people may feel embarrassed or reluctant to engage if they are not computer literate. Some intervention facilitators said they were “scared” of technology but had phones and computer tablets, although they did not use them in sessions with clients. The intervention facilitator from consultation 3 said that there were participants they had delivered PRIDE to who benefited from the paper-based version of the intervention but who may not have agreed to take part if it were presented using a web-based platform, as the use of technology would be a barrier. However, they also reasoned that even if participants were not familiar with technology, they might be willing to try the right adviser. Consultees with dementia highlighted the importance of social interactions in the delivery of interventions, stating that “people should not be replaced by computers.”

Intervention facilitators raised other considerations related to technology that may disrupt delivery of the intervention, including practical issues such as the internet either not being available or working in people’s homes, paying for internet access, and the person forgetting to charge devices. However, all consultees with dementia reported using different types of technology in their daily lives to send and receive emails, search for information, watch videos, and play games, in contrast to the expectations of the intervention facilitator on computer use among this client group.

#### Design and Accessibility Features

The designs presented include samples of text, proposed website page layouts, colors, and images. The intervention facilitators participating in consultation 3 said it was important for the design and layout of the website to be simple and felt that the wireframes fulfilled this requirement well. The supporter participating in consultation 2 felt the colors needed to be brighter to make content more noticeable, commenting that “in older age eyesight isn’t as good.” They also suggested making all text, buttons, and icons that were supposed to be clicked the same color to differentiate from content without hyperlinks to other pages on the website. Some consultees with dementia had trouble identifying the meanings behind some of the images selected to represent themes, for example, a running stick figure to represent “keeping physically active.”

Consultees said that black text on a white or yellow background would be the clearest to read, and certain colors carried certain meanings. For example, red is viewed as a danger. They felt that the colors presented on the wireframes were sufficiently clear. Consultees also expressed a preference for capital letters, followed by small print in text, rather than text presented in block capital. In terms of text size, consultees said that text might be too small if viewed on a mobile device and talked about the ability to change or set a particular text size on the website. Consultees felt it was a good idea to have audio-recorded versions of the text presented on the website pages for those who did not or could not read the content.

#### Beta Version

Following the initial consultations, Ayup iterated the website’s wireframes to incorporate several learnings. Specific developments to enhance user experience included the following:

- An option to download certain parts of the site or content for further reading offline or for printing
- An option to include a font size choice when setting up the users’ profile
- Avoiding “pop ups” that are unclear
- Changing design styles too much to keep consistency
- Prioritizing contact via a phone call when using the log-in system
- Removing block capitals and keeping all words in sentence case
- Making clickable buttons more obvious
- Placing a title next to icons so there is less ambiguity

The ability to skip through the steps in the first PRIDE session was identified as something to be modified. A linear process, by which users had to complete a sequence of 26 steps in the same order (before being able to freely navigate through the content of the website), was chosen to standardize the first session of the intervention and ensure that all compulsory activities were completed. However, the consultees felt that this made the process too lengthy and having so many steps was confusing. The intervention facilitators added that this structure might also impede their ability to tailor information to the person, which they felt was an important aspect of delivering the intervention. According to a suggestion by a consultee, “Next” buttons were added at the top of each page so that pages can be bypassed if required and “Back” buttons were added so that users can move freely between the steps according to their preference. An overall action point was to review navigation across all aspects of the website to ensure all hyperlinks connect to the correct page and refine the user journey through the “plan, do, review” content, as some of the consultees noted navigation through this information felt “circular.”

A point of frustration was that the error messages displayed when data entered into the website were not accepted or when boxes requiring data were left blank. It was not specified why the data had been rejected or which aspects of the required data on the page were missing, so it was decided that all error messages should specifically and clearly reference the issue and the location of the issue.

The PRIDE intervention manual includes a series of “case stories,” demonstrating how people have overcome the challenges associated with living with dementia. These are part of the tailored content of the intervention; thus, not all case stories will be relevant to every person receiving the intervention; rather, the intervention facilitators will select case stories they think will be helpful for the person. The intervention facilitators suggested instead of being embedded in the content of the website, which may make them difficult to locate, case stories should feature in a “narrative index,” which the intervention facilitators could refer to, to make the process of picking out examples more streamlined.

Consultees were able to easily use the log-in system. However, it was suggested that to save time, intervention facilitators should be able to register the person with an account for the website before the first session, rather than as part of the first session.

### Phase 2: Development of the Final PRIDE App

#### Overview

The researchers discussed their consultations and agreed on a series of action points that were then provided to Ayup. The priority of amendments was negotiated using the MoSCoW prioritization framework based on the assumed importance and estimated time they would take to complete.

Through sprint work and user testing, the PRIDE app was refined and made as relevant to its target users as possible. The PRIDE app is a web-based app that is accessed through a web link rather than an app store logo. After modification and refinement, the PRIDE app became a functioning web-based handbook.

#### Log-in Process

**Figure 2:**
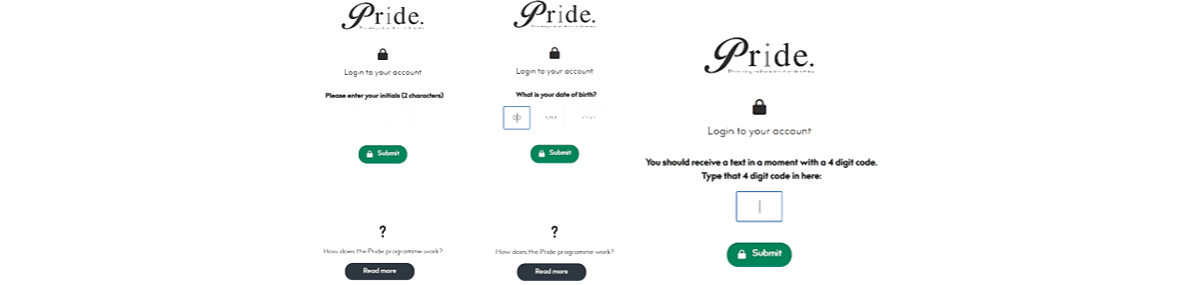
The 3-stage log-in process participants use to access the Promoting Independence in Dementia (PRIDE) app.

Facilitators create an account for individual users using 2 initials, a date of birth, and a 4-digit code that is sent to a contact number. When users log into the app, the code is sent through either SMS text messages or voice messages, and thus is accessible to those without a mobile phone. Figure 2 shows the 3-stage log-in process.

#### Introductory Session

**Figure 3:**
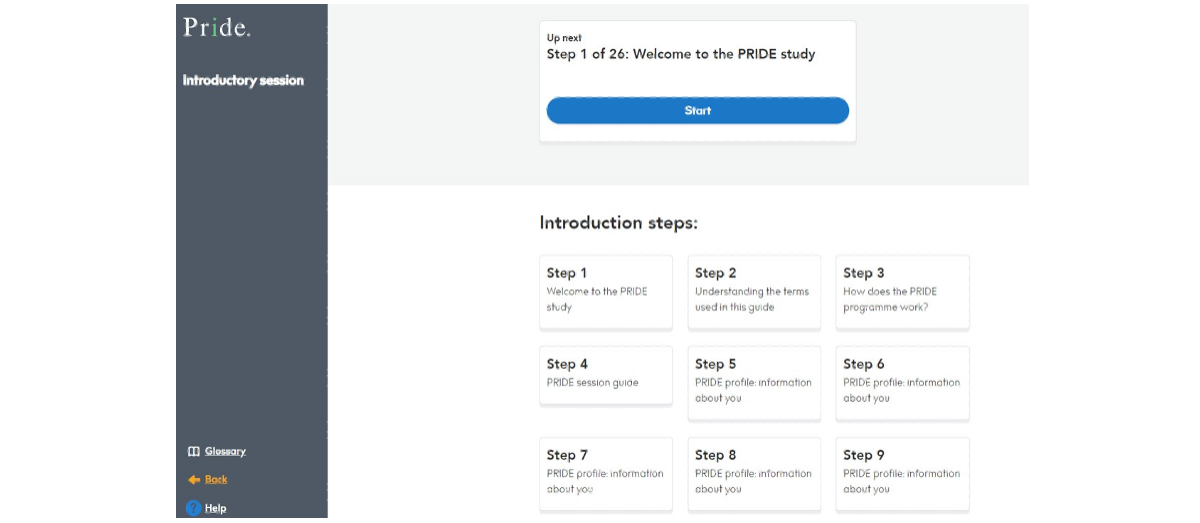
After logging on for the first time, users see the introductory session contents page. PRIDE: Promoting Independence in Dementia.

**Figure 4:**
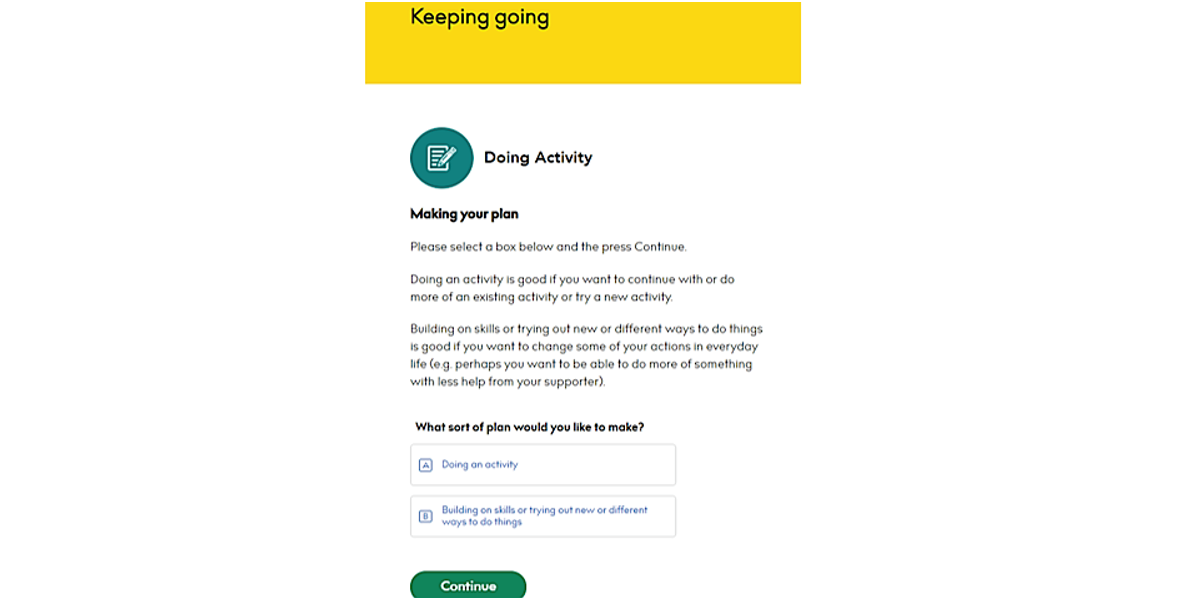
Example of the interactive activities for users to complete. The instructions were added during the sprint development.

**Figure 5:**
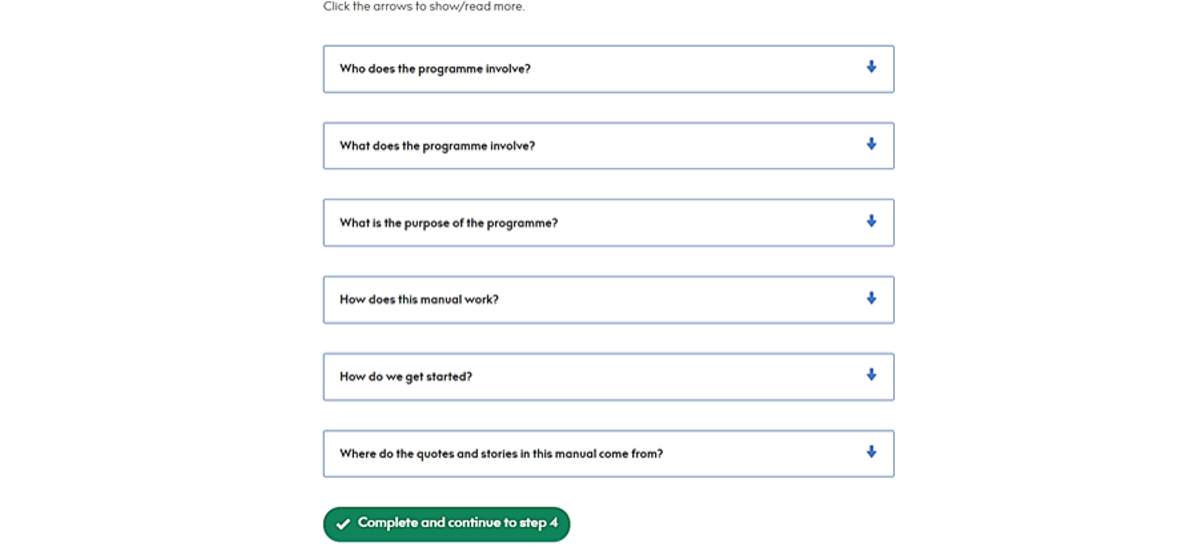
Following sprint work, collapsible sections of text were created. The arrows allow users to expand and collapse the sections as they wish.

The session structures are the same, with advisers and users completing the same introductory session as in the paper version. After logging on for the first time, users are shown 26 different steps that constitute the session content. They can save their progress and exit the app at any time, with their next step highlighted at the top of the page when they log back. A navigation bar on the left of the screen shows the users which section they are currently in. Figures 3-5 show some of the introductory session content.

#### Main Dashboard

**Figure 6:**
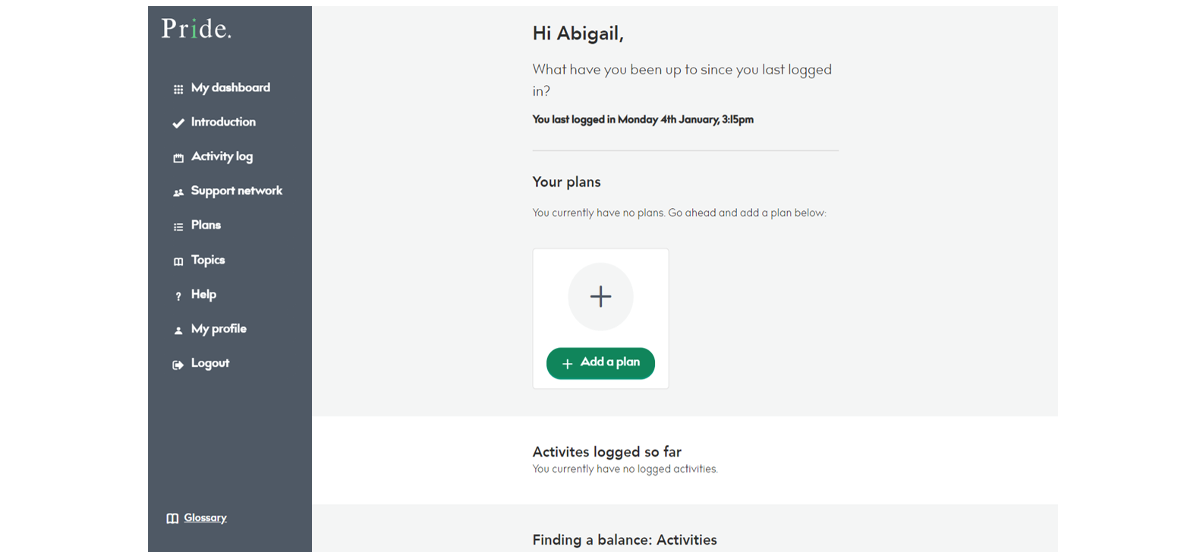
Promoting Independence in Dementia (PRIDE) app home page, where users can see their plans, activities, and access topic information.

Once the introductory steps are completed, users are transferred to the PRIDE app home page interface (Figure 6). Here, they can navigate back to the introductory session, access the individual topic areas, add members to their supporter network, create further action plans, and update their activity log.

#### PRIDE App Topics

**Figure 7:**
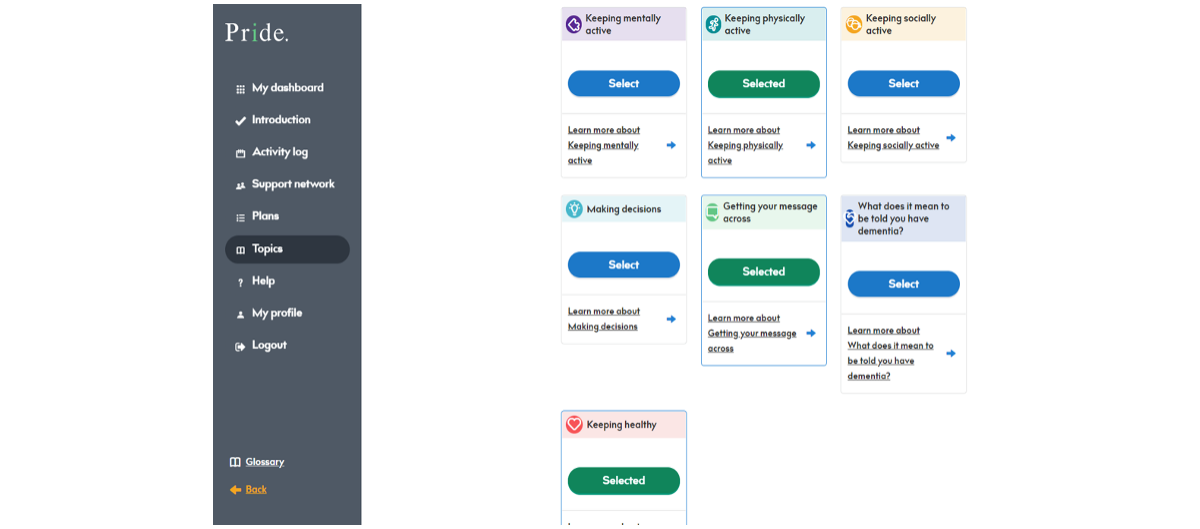
Topics section. Selecting and deselecting choices enable users to change their priorities. PRIDE: Promoting Independence in Dementia.

Participants can view information on the 7 main topics included in the PRIDE app at any time. During the introduction session, users are asked to select 3 topics that they would like to focus. This selection can be amended by users at any point through the topics section on the PRIDE app (Figure 7).

**Figure 8:**
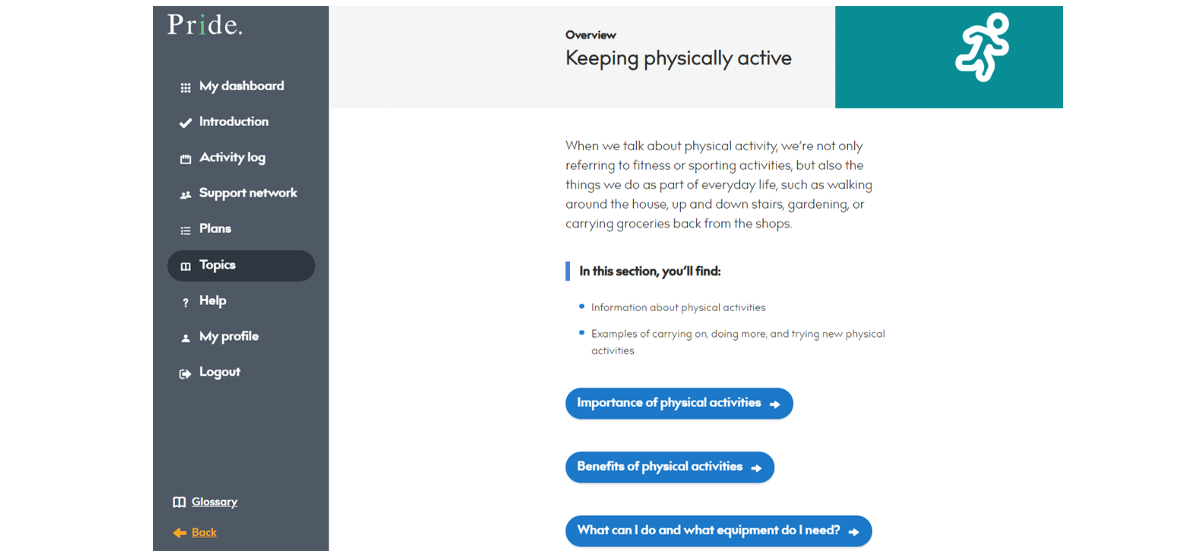
Example of a topic page. PRIDE: Promoting Independence in Dementia.

Users can also use this section to learn more about each topic. There are personal stories intertwined throughout the content to provide users with insight and reassurance of how others with dementia have made positive changes across topics. Figure 8 shows an example of one of the topic pages. Users can read all of the content or access specific subsections directly.

#### Creating Activity Plans

**Figure 9:**
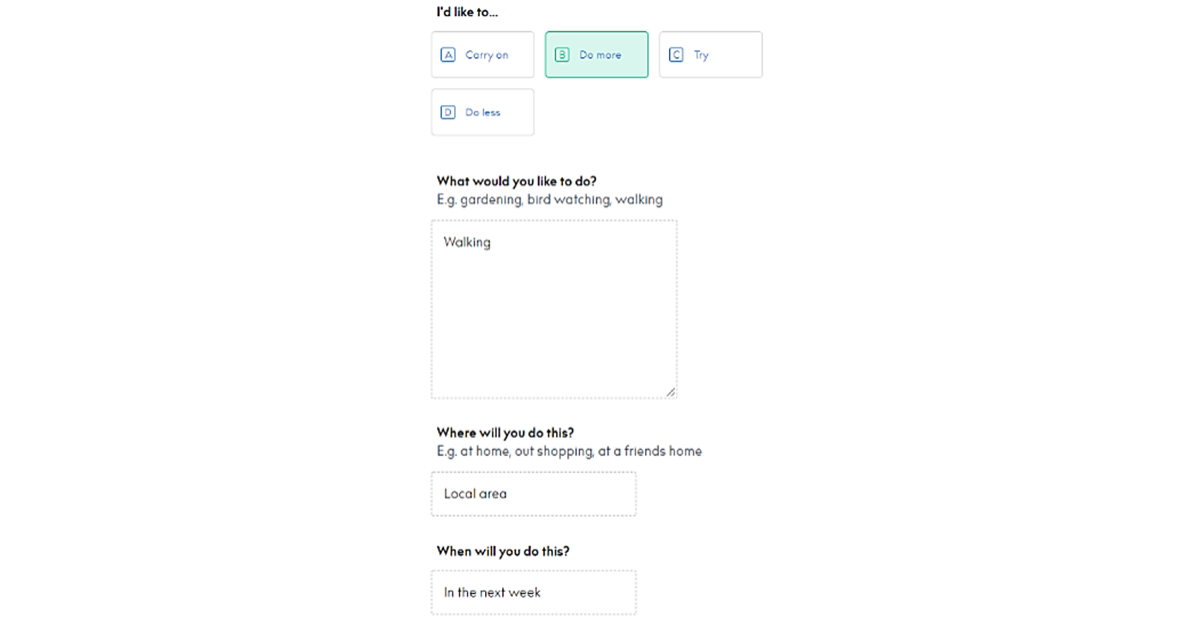
Example of creating a plan.

From the *Plans* section, users click *“*+Add a plan*”* and select the topic for which they would like to create a plan. The topic selection given on the screen are the 3 topic*s* users have selected to focus on. After selecting a topic, a page will appear asking users whether they would like to learn more about the topic or create a plan. Participants filled in the plan and selected whether they would like to carry on, try, do more, or do less of an activity. They can write about where they can execute this activity, facilitators, and potential barriers. Once completed, they click on “Save and submit plan” (Figure 9).

#### Logging an Activity

**Figure 10:**
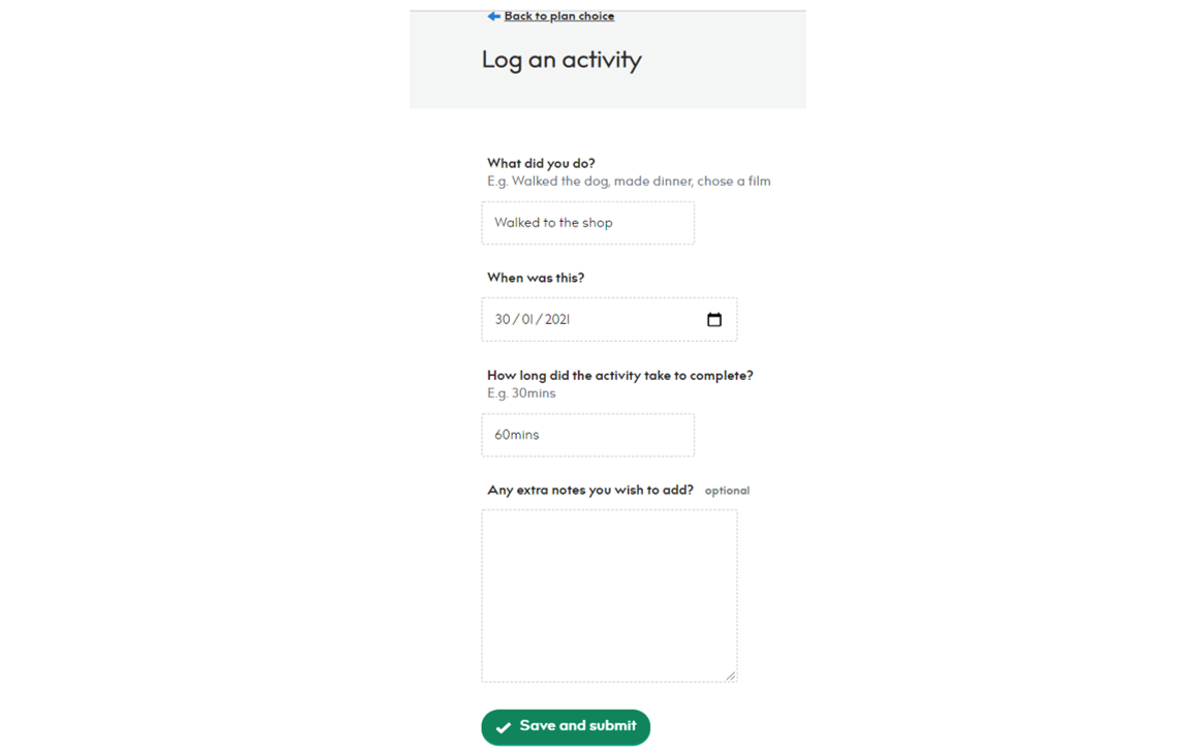
Example of logging an activity.

Once plans are created and saved, they appear on the user’s home dashboard. They select the plan they would like to log an activity. On the next page, they fill in what activity they completed, when, and how long the activity took. Clicking “Save and submit” will add that activity to their log on their dashboard (Figure 10).

#### Review Sessions

**Figure 11:**
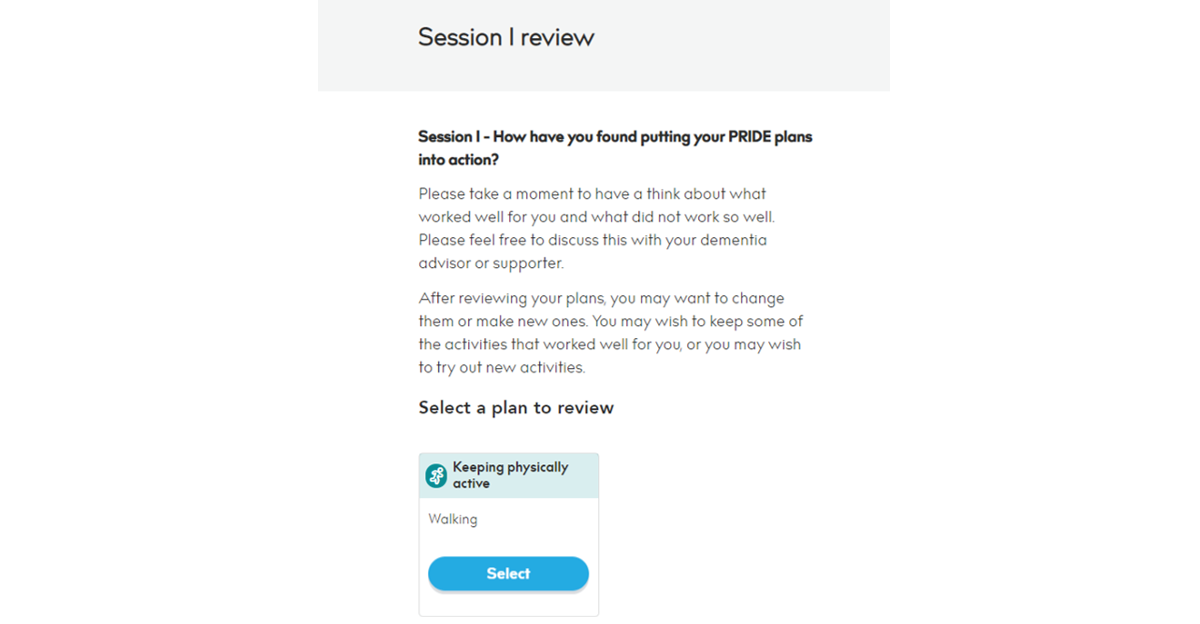
Example of selecting a plan to review. PRIDE: Promoting Independence in Dementia.

Following the introductory content, and after they have had time to use the PRIDE app in their daily lives, users completed *2* review sessions with a facilitator. From the home dashboard, users click on the begin review link and confirm that a facilitator is present. Once confirmed, the app will ask which of their plans they would like to review (Figure 11). One plan can be reviewed at a time, but the review process can be completed for as many plans as desired. The app asks users to complete boxes on how the activity went, whether anything helped or hindered them, and what the next stages were. At the bottom of the review page, participants are asked whether they would like to leave the plan as it is, revise it, or archive it (if they are happy and feel like they have completed their plan).

## Discussion

### Principal Findings

This paper presents the development of the PRIDE app, a psychosocial intervention that targets multiple domains often affected following a dementia diagnosis. Developments to enhance the dementia friendliness of the app were achieved through collaborative sprint work and the involvement of people living with dementia. To our knowledge, the PRIDE app is unique in its content, and this is the first study to present such an intervention.

### Comparison With Prior Work

A previous study on the individual Cognitive Stimulation Therapy (iCST) app helped inform our development process [10]. They took an iterative approach to app development and involved people living with dementia and their caregivers to improve the structured cognitive stimulation application. Through interviews and focus groups, the researchers were able to incorporate participant feedback into their 3 developmental sprints and explore the initial experiences of using the computerized cognitive stimulation program [10]. The iCST app was similar to the PRIDE app in that it was a one-to-one program delivered at home on a touchscreen tablet. However, the interventions differed as iCST was carer-led, only applicable to tablets, and purely focused on cognitive stimulation activities. Although there were differences, the iterative approach used was very similar to that in the PRIDE app development, as feedback from people with dementia and their supporters also informed the sprint work [10]. In addition, the discussion guide for their interviews helped inform the questions asked when gathering feedback and for the interview that will be conducted with participants following their use of the PRIDE app.

Over the last decade, there have been changes in the quantification of health and quality of life. Some have proposed an update to the World Health Organization’s definition of health by altering the focus on how well an individual can self-manage and adapt to physical, mental, and social health challenges [11]. The PRIDE app aims to meet this evidence gap by providing an intervention that covers multiple domains relevant to the revised World Health Organization definition and targets a range of self-management concepts.

A previous systematic review of web- and app-based interventions for dementia showed their potential to produce positive outcomes in self-management and can be successfully delivered through a range of methods [6]. Existing interventions targeted several self-management concepts, such as independence and activities of daily living, but there was an inconsistency in which domains often affected by dementia were targeted by interventions, and some purely focused on 1 concept. The review also revealed a lack of high-quality evidence on these types of dementia interventions and no studies researching an intervention that encompasses physical, cognitive, social, and emotional domains. The PRIDE app aims to meet this evidence gap by providing an intervention that covers multiple domains and targets a range of self-management concepts.

### Limitations

We did not foresee the low recruitment of user-testing volunteers, and this delay had a wider impact on the study timelines. Despite the call for volunteers to go out to local and national groups, there was very limited interest in user testing. This was likely because of the COVID-19 pandemic, the change in people’s priorities, and lack of interest in research. However, the recruited volunteers were experienced in dementia studies and provided useful feedback. Another limitation is the removal of the field-testing phase. Originally, this stage was to be incorporated following the second sprint to assess the PRIDE app’s usability and accessibility, with a third sprint proposed to resolve any urgent problems. A delayed field-testing phase was not a viable option for this study because of the time constraints and resources available for the study.

The COVID-19 pandemic meant that remote working was necessary for the vast majority of the PRIDE app development. It also required community and PPI groups for people living with dementia and their families to either close temporarily or move on the web. Unfortunately, these required measures contributed to difficulties in finding user-testing volunteers and removing an accessible source of feedback during the ongoing app development. Delays caused by these difficulties led to the second sprint, which had a knock-on effect on the rest of the study timelines. As diagnoses were not recorded from those involved in the development of the app, conclusions about specific types of dementia were limited. This should be considered when conducting future research to understand any potential barriers specific dementias could cause.

Following the development work, the PRIDE app will be the focus of the reach, effectiveness, adoption, implementation, and maintenance (RE-AIM) study [12]. The app offers people living with dementia a central source of information and support on a range of domains commonly affected by dementia; and this study will explore the potential reach, effectiveness, and adoption of the intervention. Although a larger trial will be needed to assess the potential effectiveness more comprehensively, the RE-AIM study will provide initial insight into whether the PRIDE app could be a feasible intervention, suitable for further research, and whether it could have positive outcomes for people with dementia and their families.

### Conclusions

The PRIDE app has evolved from its initial prototype [5] into a more dementia-friendly and usable program that is of a standard suitable for wider testing. It has the potential to advance the previous evidence into web- and app-based interventions, in addition to providing better support for self-management, improving individuals’ level of independence, and enhancing the quality of life of people with dementia and their families. The finished version will be tested in a RE-AIM study, with its potential reach, effectiveness, and adoption explored. This study will contribute further to the evidence base and our understanding of how web- and app-based interventions could be successfully implemented in dementia management. Feedback gathered during the RE-AIM study will lead to any further developments to the app to increase its applicability and usability to the target audience, such as considering alternative log-in methods and identifying barriers for specific dementia types. It will also provide further understanding of the barriers and facilitators that have a significant impact on the adoption of these interventions and how these could be overcome in future research.

## Abbreviations

iCST: individual Cognitive Stimulation Therapy
MoSCoW: Must Have, Should Have, Could Have, and Will Not Have this time
PPI: Patient and Public Involvement
PRIDE: Promoting Independence in Dementia
RE-AIM: reach, effectiveness, adoption, implementation, and maintenance
UoN: University of Nottingham
